# Patients with Parkinson's disease demonstrate deficits in visual‐spatial memory in the Chinese Visual Retention Test

**DOI:** 10.1002/brb3.3345

**Published:** 2023-12-31

**Authors:** Chunyan Liu, Meng Yuan, Songbin He

**Affiliations:** ^1^ Department of Critical Care Medicine Huzhou Central Hospital Huzhou China; ^2^ Department of Neurology Zhoushan Hospital, Wenzhou Medical University Zhoushan China

**Keywords:** Parkinson's disease, visual‐spatial memory, the Chinese Visual Retention Test

## Abstract

**Objective:**

We aimed to explore the existence of visual‐spatial memory deficit in patients with Parkinson's disease (PD) without dementia in the Chinese Visual Retention Test, as well as to assess whether their performance is related to age, duration, severity, stage, and dopamine (DA) dose.

**Methods:**

Forty‐two patients with PD and 30 healthy controls were included in our study. The Chinese Visual Retention Test was used to evaluate the visual‐spatial memory of the subjects. Parameters of the Chinese Visual Retention Test were compared between the two groups. Correlation analysis and multiple linear regression analysis were used to explore the associations of the Chinese Visual Retention Test with age, duration, severity, stage of PD, and DA dose.

**Results:**

Three correct scores in the Chinese Visual Retention Test were all significantly lower in the PD group than in the control group. The total error scores, error scores of omissions, deformation, and persistence in the PD group were significantly higher than those in the control group. Correlation analysis showed the total error scores in the Chinese Visual Retention Test was positively correlated with UPDRS III score and H‐Y classification. Multiple linear regression analysis showed that the total error scores in the Chinese Visual Retention Test were associated with the UPDRS III score and H‐Y classification.

**Conclusion:**

Patients with PD without dementia had visual‐spatial memory deficits in the Chinese Visual Retention Test which may be affected by the severity and clinical stage of PD.

## Graphical abstract

The Chinese Visual Retention Test was used to evaluate the visual‐spatial memory of 42 patients with PD and 30 healthy controls. Our study found that patients with PD without dementia had visual‐spatial memory deficits in the Chinese Visual Retention Test which may be affected by the severity and clinical stage of PD.

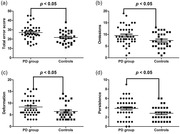

## 1 INTRODUCTION

Parkinson's disease (PD) is a common degenerative disease of the central nervous system. PD is currently defined as the presence of bradykinesia combined with either rest tremor, rigidity, or both (Bloem et al., 2021). Cognitive dysfunction is one of the most prevalent non‐motor symptoms of PD (Aarsland et al., 2017; Baiano et al., 2020; Roheger et al., 2018). As PD is predominantly a disorder in the elderly, it is not surprising that patients in this age group often exhibit a decline in cognitive function. Worsening cognitive function can often be debilitating for patients and their caregivers (Lawson et al., 2016).

Visual‐spatial memory abilities have been reported to predict progressive cognitive impairment and PD dementia (Yang et al., 2018). Several previous studies (Del Pino et al., 2021; Fernandez‐Baizan et al., 2020; Netherton et al., 1989) have reported that patients with PD present significant impairment in visual‐spatial memory abilities. However, the evaluation of the visual‐spatial memory abilities in patients with PD has not been consistent.

The Benton Visual Retention Test was developed in 1955 and has been widely used to evaluate cognitive function (Didycz et al., 2018; Talarowska et al., 2011). Further, Netherton et al. (1989) found that PD was associated with changes in visual‐spatial memory which was predicted by age. The Chinese Visual Retention Test (Tang & Gong, 1993) was translated and adapted from the Benton Visual Retention Test, which details the implementation methods, scoring principles, validity, and reliability.

Our study explored the existence of visual‐spatial memory impairment in patients with PD without dementia using the Chinese Visual Retention Test, as well as assessed whether their performance is related to their age, duration, severity, stage of PD, and dopamine (DA) dose.

## 2 MATERIALS AND METHODS

### 2.1 Subjects

This case‐control study was conducted between October 2015 and July 2017 at the Department of Neurology of Zhoushan Hospital. Forty‐two patients with PD and 30 age‐matched healthy controls were included. Written informed consent was obtained from all subjects. The study was approved by the Ethics Committee of Zhoushan Hospital (Liu et al., 2017). Forty of the patients with PD were treated with levodopa or a DA agonist. PD was diagnosed according to the Movement Disorder Society (MDS) Clinical Diagnostic Criteria for PD (Postuma et al., 2015). The exclusion criteria were patients with (1) dementia, severe anxiety, depression, or psychosis, (2) structural damage in the retina or ophthalmologic disease, and (3) other severe diseases, such as cancer, heart failure, renal failure, and chronic obstructive pulmonary disease.

## 3 METHODS

All enrolled patients with PD underwent a series of detailed neurological examinations, including brain magnetic resonance imaging (MRI) examination, routine blood tests, assessment of biochemical function, and levels of myocardial enzymes. Hamilton Anxiety and Depression Scale was used to evaluate the psychological state, and the Mini‐Mental State Examination (MMSE) was used to assess general cognition. The severity of PD was assessed using the motor part of the original unified Parkinson's disease rating scale (UPDRS III), and the PD stage was assessed using the Hoehn‐Yahr (H‐Y) classification (Liu et al., 2017). Patients were assessed in the “on” state before the morning dose of the drug. The DA dose, also known as levodopa‐equivalent dose (LED), was calculated using the formula (Tomlinson et al., 2010). Ophthalmologic evaluations were performed at the eye clinic and included a visual acuity test (Snellen table), an Ishihara color test, biomicroscopy, and intraocular pressure measurement using the Goldmann applanation tonometer (Liu et al., 2017). Optical coherence tomography (OCT) was used to evaluate the retinal structure.

The Chinese Visual Retention Test evaluation includes three different series: Series C, Series D, and Series E. Each series includes 10 cards, each of which has one or more graphics. In this study, methods C, B, and D were used sequentially for each series, of which the correct and error points were recorded. There are six types of errors: omission, deformation, persistence, rotation, dislocation, and incorrect size. Each type of error is scored as one point to determine the total error score.

The SPSS software (Inc. Chicago, IL, USA; version 21.0) was used for data analysis. Variables with normal distributions were presented as the mean ± standard deviation and were compared with independent samples *t*‐tests. Non‐normally distributed variables and low numbers were expressed as median (25th to 75th percentiles), which were compared with the Mann–Whitney *U* test. The chi‐square test was used to determine group distribution. Correlations of the Chinese Visual Retention Test with age, duration of PD, UPDRS III scores, and DA dose were evaluated using Pearson's correlation test. The correlation of the Chinese Visual Retention Test with the H‐Y classification was evaluated using the Spearman's rank correlation test. Multiple linear regression analysis was used to assess the association of the Chinese Visual Retention Test with age, duration of PD, UPDRS III score, H‐Y classification, and DA dose.

## 4 RESULTS

### 4.1 Quantitative analysis of participants

We examined 42 patients with PD and 30 age‐matched healthy controls. The demographic and clinical assessment data of patients with PD and healthy controls are presented in Table 1. The groups were not statistically different in age (*t*‐test: *t* = 0.25, *p* = .803), sex distribution (chi‐square test: χ^2^ = 0.002, *p* = .968), mean body mass index (*t*‐test: *t* = −0.05, *p* = .957), and years of education (*t*‐test: *t* = 0.192, *p* = .867) (Liu et al., 2017). All patients with PD were between H‐Y stages one and four (median, 2.00).

**TABLE 1:** Demographic and clinical data of subjects.

	PD	Control	*p*
*N*	42	30	
Sex (male/female)	18/24	13/17	.968
Age (years)	69.24 ± 6.94	68.83 ± 6.54	.803
BMI	21.28 ± 2.51	21.32 ± 2.94	.957
Education (years)	4.00 ± 3.57	3.83 ± 3.72	.867
Duration of PD (years)	3.46 ± 2.47	–	
UPDRS III score	10.50 ± 6.78	–	
H‐Y classification	2.00 (1.50, 2.50)	–	
DA dose (mg)	271.26 ± 163.24	–	

Abbreviations: BMI: body mass index; DA: dopamine; H‐Y: Hoehn and Yahr scale; PD: Parkinson's disease; UPDRS: unified Parkinson's disease rating scale part III.

### 4.2 Abnormality in the Chinese Visual Retention Test

**FIGURE 1:**
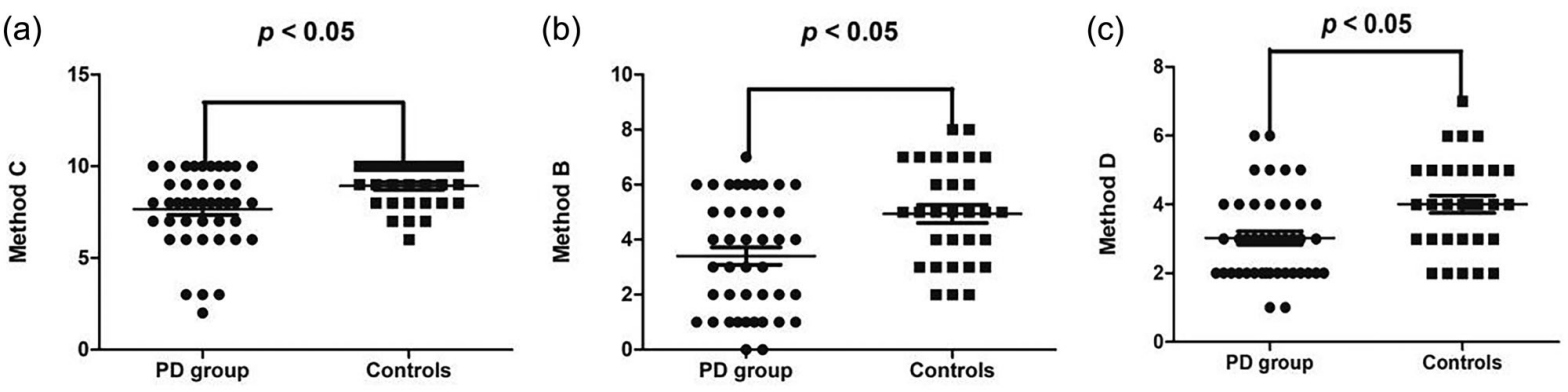
(a) Comparison of the correct scores of method C between the PD group and the controls. (b) Comparison of the correct scores of method B between the PD group and the controls. (c) Comparison of the correct scores of method D between the PD group and the controls.

**FIGURE 2:**
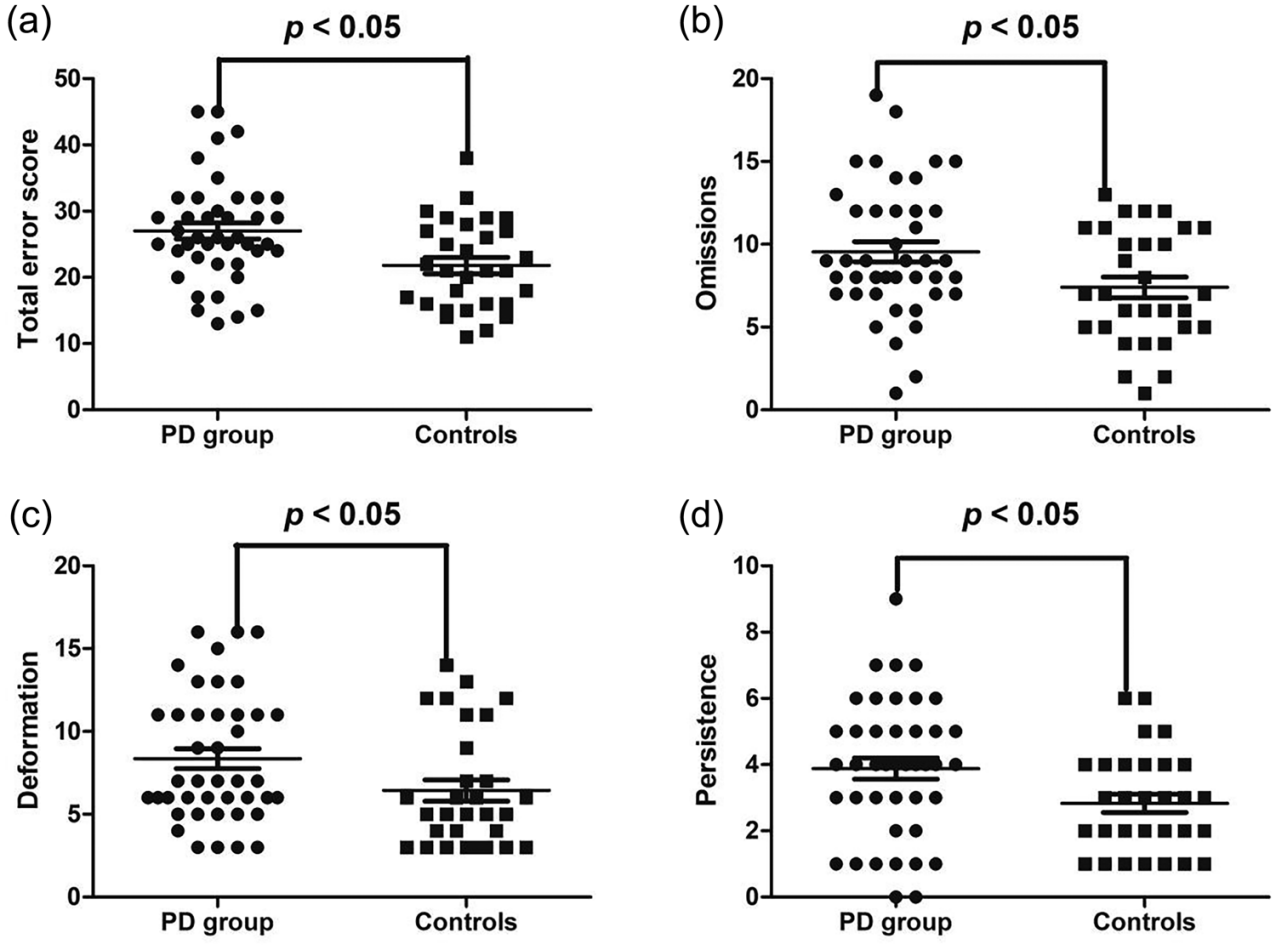
(a) Comparison of the total error scores of the Chinese Visual Retention Test between the PD group and the controls. (b) Comparison of omission error scores between the PD group and the controls. (c) Comparison of deformation error scores between the PD group and the controls. (d) Comparison of persistence error scores of persistence between the PD group and the controls.

The results of the Chinese Visual Retention Test showed that the correct scores for methods C, B, and D were all significantly lower in the PD group than in the control group (*U* = 397.5, *Z* = −2.729, *p* = .006; *t* = −3.244, *p* = .002; *t* = −3.132; *p* = .003, respectively) (Table 2 and Figure 1). The total error scores, as well as error scores of omissions, deformation, and persistence were significantly higher in the PD group than in the control group (*t* = 2.972, *p* = .004; *t* = 2.402; *p* = .019; *t* = 2.148, *p* = .035; *t* = 2.345; *p* = .022, respectively) (Table 2 and Figure 2). There were no significant differences in the error scores of rotation, dislocation, and incorrect size between the two groups (*p* all > .05).

**TABLE 2:** Intergroup comparison of the Chinese Visual Retention Test.

	PD	Control	*p*
Correct scores of method C	3 (0, 4)	1 (0, 2)	.006**
Correct scores of method B	3.40 ± 2.07	4.93 ± 1.82	.002**
Correct scores of method D	3.03 ± 1.26	4.00 ± 1.36	.003**
Total error scores	27.02 ± 7.78	21.8 ± 6.70	.004**
Omission error scores	9.55 ± 3.96	7.40 ± 3.40	.019*
Deformation error scores	8.36 ± 3.88	6.43 ± 3.55	.035*
Persistence error scores	4 (3, 5)	3 (2, 4)	.022*
Rotation error scores	2 (2, 4)	2 (1, 3)	.234
Dislocation error scores	1 (0, 2)	1 (0, 2)	.787
Incorrect size error scores	1 (0, 2)	2 (1, 2)	.363

*p < .05.

**p < .01.

Abbreviation: PD: Parkinson's disease.

### 4.3 The correlation of the Chinese Visual Retention Test with PD

**FIGURE 3:**
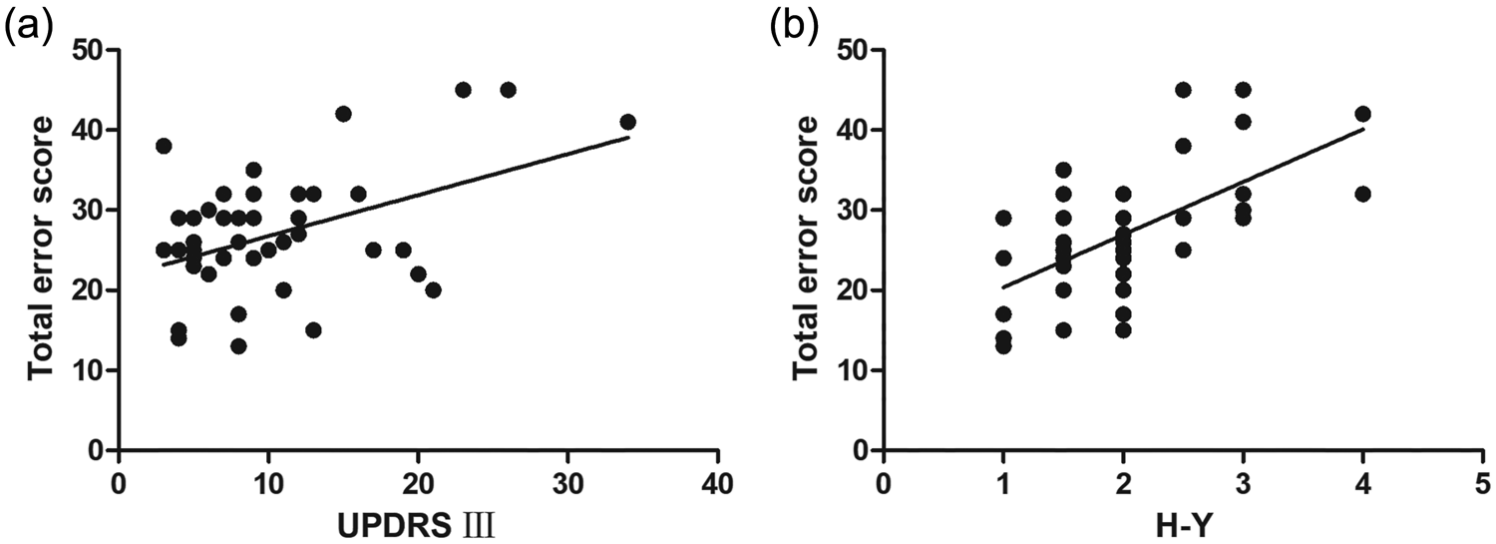
(a) Correlation of the total error scores of the Chinese Visual Retention Test and UPDRS III scores in PD group. (b) Correlation of the total error scores of the Chinese Visual Retention Test and H‐Y classification in PD group.

In the PD group, the total error scores in the Chinese Visual Retention Test were positively correlated with the UPDRS III score (*r* = 0.446, *p* < .01) (Figure 3) and H‐Y classification (*r*_s_ = 0.637, *p* < .01) (Figure 3), but not with age, PD duration, and DA dose. The result of multiple linear regression analysis showed that the total error scores in the Chinese Visual Retention Test were associated with the UPDRS III score (*B* = 0.367, *p* < .05, Table 3) and H‐Y classification (*B* = 7.258, *p* < .01, Table 3).

**TABLE 3:** Multiple linear regression analysis of factors associated with the Chinese Visual Retention Test.

Dependent variable	Unstandardized coefficient	*t*	*p*	95% CI	
	*B*	SE			
Age	–0.073	0.157	–0.465	0.645	–0.392 to 0.246
PD duration	0.830	0.555	–1.495	0.144	–1.957 to 0.296
UPDRS‐III	0.367	0.168	2.182	0.036*	0.026 to 0.708
H‐Y	7.258	1.604	4.526	0.000**	4.006 to 10.510
DA dose	–0.003	0.008	–0.383	0.704	–0.019 to 0.013

*p < .05.

**p < .01.

Abbreviations: CI: confidence interval; DA: dopamine; H‐Y: Hoehn and Yahr scale; PD: Parkinson's disease; SE: standard error; UPDRS: unified Parkinson's disease rating scale part III.

## 5 DISCUSSION

Although previous studies on visual‐spatial memory in PD are scarce, some have shown that patients express subjective complaints in their daily spatial activities, such as reading a map, using navigation devices, or finding their way while driving (Davidsdottir et al., 2005). Our findings suggest that patients with PD without dementia already have visual‐spatial memory deficits according to the Chinese Visual Retention Test, which was related to the severity of motor symptoms and clinical stage of PD.

Older age is considered a significant predictor of poor cognitive performance in PD patients (Hu et al., 2014; Oikonomou et al., 2021; Vingerhoets et al., 2003). One possible reason is that age affects the progress of PD, and older patients tend to progress quickly and can rapidly develop into dementia. Contrary to these studies, our study showed no association between visual‐spatial memory deficits and age. Age itself may not be an independent risk factor for visual‐spatial memory deficit. In agreement with previous studies (Oikonomou et al., 2021; Shuncai et al., 2016), our study also suggest PD duration and DA dose are not independent risk factors for visual‐spatial memory deficit.

The etiology of PD is multifactorial, and progressive loss of dopaminergic neurons in the substantia nigra is widely accepted. However, DA appears to be more involved than the substantia nigra. Information processing, such as planning, problem‐solving, decision‐making, and response selection, is associated with the function of frontostriatal circuits. Recent studies have attempted to link cognitive symptoms with specific neuronal circuits in the basal ganglia and its connections. Cognitive deficits in PD may be linked to a malfunction of frontal‐basal ganglia neural circuits, which are important for executive functions. Nevertheless, frontal‐basal ganglionic dysfunction only partially accounts for the visual‐spatial memory deficits present in PD (Liu et al., 2015). It has been proposed that the loss of DA in the substantia nigra and striatum affects an extensive neural network, including prefrontal structures, via mesolimbic‐frontal fibers. All these factors may contribute to visual‐spatial memory deficits and progressive cognitive dysfunction in PD patients.

Given the high prevalence rates of dementia in PD, prediction of the risk of its progression seems highly important. However, there are lack of effective early biomarkers for the diagnosis of dementia in PD. Our study suggests that visual‐spatial memory deficits occur in the early stages of cognitive impairment in patients with PD. As cognitive impairment appears in the early stages of PD, the guidelines for PD‐mild cognitive impairment (PD‐MCI) (Goldman et al., 2013) complement the current criteria defining PD. Deficits in visual‐spatial skills can be considered a prodromal symptom of PD (Kehagia et al., 2013). The results of our study also suggest that visual‐spatial memory impairment may be a predictor of progression of cognitive impairment and that visual‐spatial memory tests may be beneficial for the diagnosis of PD.

The limitations of our study are as follows: (1) Our study included a small number of patients with PD and controls, which may not conclusively support our findings. (2) PD patients with retinal impairment were excluded; therefore, we were unable to determine the relationship between retinal impairment and deficits in visual‐spatial memory.

## 6 CONCLUSION

Patients with PD without dementia already had visual‐spatial memory deficits in the Chinese Visual Retention Test. Further, impaired visual‐spatial memory may be affected by the severity and clinical stage of PD.

## AUTHOR CONTRIBUTIONS

Songbin He conceived and designed the study. Chunyan Liu contributed to the study design and completion, and data management. Meng Yuan performed statistical analyses. Chunyan Liu drafted the manuscript and Songbin He reviewed and edited the manuscript. All authors have read and approved the final manuscript.

## CONFLICT OF INTEREST STATEMENT

The authors declare that there is no conflict of interest regarding the publication of this paper.

### PEER REVIEW

The peer review history for this article is available at https://publons.com/publon/10.1002/brb3.3345.

## Data Availability

Data can be obtained from the corresponding author by email.

## References

[brb33345-bib-0001] Aarsland, D. , Creese, B. , Politis, M. , Chaudhuri, K. R. , Ffytche, D. H. , Weintraub, D. , & Ballard, C. (2017). Cognitive decline in Parkinson disease. Nature Reviews. Neurology, 13(4), 217–231. 10.1038/nrneurol.2017.27 28257128 PMC5643027

[brb33345-bib-0002] Baiano, C. , Barone, P. , Trojano, L. , & Santangelo, G. (2020). Prevalence and clinical aspects of mild cognitive impairment in Parkinson's disease: A meta‐analysis. Movement Disorders: Official Journal of the Movement Disorder Society, 35(1), 45–54. 10.1002/mds.27902 31743500

[brb33345-bib-0003] Bloem, B. R. , Okun, M. S. , & Klein, C. (2021). Parkinson's disease. Lancet (London, England), 397(10291), 2284–2303. 10.1016/S0140-6736(21)00218-X 33848468

[brb33345-bib-0004] Davidsdottir, S. , Cronin‐Golomb, A. , & Lee, A. (2005). Visual and spatial symptoms in Parkinson's disease. Vision research, 45(10), 1285–1296. 10.1016/j.visres.2004.11.006 15733961

[brb33345-bib-0005] Del Pino, R. , Acera, M. , Murueta‐Goyena, A. , Lucas‐Jiménez, O. , Ojeda, N. , Ibarretxe‐Bilbao, N. , Peña, J. , Reyero, P. , Cortés, J. , Tijero, B. , Galdós, M. , Gómez‐Esteban, J. C. , & Gabilondo, I. (2021). Visual dysfunction is associated with cognitive impairment in Parkinson's disease. Parkinsonism & related disorders, 92, 22–25. 10.1016/j.parkreldis.2021.10.005 34662807

[brb33345-bib-0006] Didycz, B. , Nitecka, M. , & Bik‐Multanowski, M. (2018). The use of d2 and Benton tests for assessment of attention deficits and visual memory in teenagers with phenylketonuria. JIMD Reports, 40, 23–29. 10.1007/8904_2017_60 28940169 PMC6122018

[brb33345-bib-0007] Fernandez‐Baizan, C. , Paula Fernandez Garcia, M. , Diaz‐Caceres, E. , Menendez‐Gonzalez, M. , Arias, J. L. , & Mendez, M. (2020). Patients with Parkinson's disease show alteration in their visuospatial abilities and in their egocentric and allocentric spatial orientation measured by card placing tests. Journal of Parkinson's Disease, 10(4), 1807–1816. 10.3233/JPD-202122 33016894

[brb33345-bib-0008] Goldman, J. G. , Holden, S. , Bernard, B. , Ouyang, B. , Goetz, C. G. , & Stebbins, G. T. (2013). Defining optimal cutoff scores for cognitive impairment using Movement Disorder Society Task Force criteria for mild cognitive impairment in Parkinson's disease. Movement Disorders: Official Journal of the Movement Disorder Society, 28(14), 1972–1979. 10.1002/mds.25655 24123267 PMC4164432

[brb33345-bib-0009] Hu, M. T. M. , Szewczyk‐Królikowski, K. , Tomlinson, P. , Nithi, K. , Rolinski, M. , Murray, C. , Talbot, K. , Ebmeier, K. P. , Mackay, C. E. , & Ben‐Shlomo, Y. (2014). Predictors of cognitive impairment in an early stage Parkinson's disease cohort. Movement Disorders: Official Journal of the Movement Disorder Society, 29(3), 351–359. 10.1002/mds.25748 24395708 PMC4235340

[brb33345-bib-0010] Kehagia, A. A. , Barker, R. A. , & Robbins, T. W. (2013). Cognitive impairment in Parkinson's disease: The dual syndrome hypothesis. Neuro‐Degenerative Diseases, 11(2), 79–92. 10.1159/000341998 23038420 PMC5079071

[brb33345-bib-0011] Lawson, R. A. , Yarnall, A. J. , Duncan, G. W. , Breen, D. P. , Khoo, T. K. , Williams‐Gray, C. H. , Barker, R. A. , Collerton, D. , Taylor, J. P. , Burn, D. J. , & ICICLE‐PD Study Group . (2016). Cognitive decline and quality of life in incident Parkinson's disease: The role of attention. Parkinsonism & Related Disorders, 27, 47–53. 10.1016/j.parkreldis.2016.04.009 27094482 PMC4906150

[brb33345-bib-0012] Liu, C. , Zhang, Y. , Tang, W. , Wang, B. , Wang, B. , & He, S. (2017). Evoked potential changes in patients with Parkinson's disease. Brain and Behavior, 7(5), e00703. 10.1002/brb3.703 28523237 PMC5434200

[brb33345-bib-0013] Liu, Z. , Uchiyama, T. , Sakakibara, R. , & Yamamoto, T. (2015). Underactive and overactive bladders are related to motor function and quality of life in Parkinson's disease. International Urology and Nephrology, 47(5), 751–757. 10.1007/s11255-015-0951-y 25792006

[brb33345-bib-0014] Netherton, S. D. , Elias, J. W. , Albrecht, N. N. , Acosta, C. , Hutton, J. T. , & Albrecht, J. W. (1989). Changes in the performance of parkinsonian patients and normal aged on the Benton visual retention test. Experimental Aging Research, 15(1‐2), 13–18. 10.1080/03610738908259753 2583210

[brb33345-bib-0015] Oikonomou, P. , Van Wamelen, D. J. , Weintraub, D. , Aarsland, D. , Ffytche, D. , Martinez‐Martin, P. , Rodriguez‐Blazquez, C. , Leta, V. , Borley, C. , Sportelli, C. , Trivedi, D. , Podlewska, A. M. , Rukavina, K. , Rizos, A. , Lazcano‐Ocampo, C. , & Ray Chaudhuri, K. (2021). Nonmotor symptom burden grading as predictor of cognitive impairment in Parkinson's disease. Brain and Behavior, 11(5), e02086. 10.1002/brb3.2086 33645912 PMC8119808

[brb33345-bib-0016] Postuma, R. B. , Berg, D. , Stern, M. , Poewe, W. , Olanow, C. W. , Oertel, W. , Obeso, J. , Marek, K. , Litvan, I. , Lang, A. E. , Halliday, G. , Goetz, C. G. , Gasser, T. , Dubois, B. , Chan, P. , Bloem, B. R. , Adler, C. H. , & Deuschl, G. (2015). MDS clinical diagnostic criteria for Parkinson's disease. Movement Disorders: Official Journal of the Movement Disorder Society, 30(12), 1591–1601. 10.1002/mds.26424 26474316

[brb33345-bib-0017] Roheger, M. , Kalbe, E. , & Liepelt‐Scarfone, I. (2018). Progression of cognitive decline in Parkinson's disease. Journal of Parkinson's disease, 8(2), 183–193. 10.3233/JPD-181306 PMC6004891 29914040

[brb33345-bib-0018] Shuncai, Y. , Leifeng, L. , Jing, C. , & Psychiatry, D. O. (2016). The evaluation of the impairments in cognition and motion etc and their influencing factors in patients with Parkinson's disease and dementia. Journal of International Psychiatry, 04, 610–612.

[brb33345-bib-0019] Talarowska, M. , Florkowski, A. , Zboralski, K. , & Gałecki, P. (2011). Results of the Benton visual retention test and the bender visual–motor gestalt test among patients suffer from depressive disorders and organic depressive disorders. Psychiatria Polska, 45(4), 495. 22232976

[brb33345-bib-0020] Tang, Q. , & Gong, Y. (1993). Research on reliability and validity of visual persistence test. Chinese Journal of Clinical Psychology, 000(002), 87–128. doi:CNKI:SUN:ZLCY.0.1993‐02‐006. https://www.nstl.gov.cn/paper_detail.html?id=f7c27449abd0eb50559638fef10c1596

[brb33345-bib-0021] Tomlinson, C. L. , Stowe, R. , Patel, S. , Rick, C. , Gray, R. , & Clarke, C. E. (2010). Systematic review of levodopa dose equivalency reporting in Parkinson's disease. Movement Disorders: Official Journal of the Movement Disorder Society, 25(15), 2649–2653. 10.1002/mds.23429 21069833

[brb33345-bib-0022] Vingerhoets, G. (2003). Predictors of cognitive impairment in advanced Parkinson's disease. Journal of Neurology, Neurosurgery, and Psychiatry, 74(6), 793–796. 10.1136/jnnp.74.6.793 12754355 PMC1738465

[brb33345-bib-0023] Yang, K. , Shen, B. , Li, D.‐K. , Wang, Y. , Zhao, J. , Zhao, J. , Yu, W.‐B. , Liu, Z.‐Y. , Tang, Y.‐L. , Liu, F.‐T. , Yu, H. , Wang, J. , Guo, Q.‐H. , & Wu, J.‐J. (2018). Cognitive characteristics in Chinese non‐demented PD patients based on gender difference. Translational Neurodegeneration, 7, 16. 10.1186/s40035-018-0120-1 30038782 PMC6052700

